# Current Practices in, Reimbursement Opinions About, and Clinical Indications of Adaptive Radiation Therapy: Results From the American College of Radiation Oncology Survey

**DOI:** 10.7759/cureus.93927

**Published:** 2025-10-06

**Authors:** Jared L Pasetsky, Dwight E Heron, Paul E Wallner, Jayden R Gracie, Christopher D Jahraus, Tarita O Thomas

**Affiliations:** 1 Radiation Oncology, Columbia University, New York, USA; 2 Radiation Oncology, Bon Secours Mercy Health, Youngstown, USA; 3 Radiation Oncology, American College of Radiation Oncology, Moorestown, USA; 4 Radiation Oncology, Vanderbilt University Medical Center, Nashville, USA; 5 Radiation Oncology, American College of Radiation Oncology, Alabaster, USA; 6 Radiation Oncology, Northwestern University Feinberg School of Medicine, Chicago, USA

**Keywords:** adaptive radiation therapy, community survey, ct-guided adaptive radiotherapy, medical billing, mri-guided linac, online adaptive radiotherapy

## Abstract

Introduction: Adaptive radiation therapy (ART) is generally defined as adjusting radiation dose delivery based on changes observed in the tumor or surrounding normal tissues during the course of treatment. Improvements in dosimetry with ART have been widely reported in the literature, in addition to the increased costs attributed to increased physician time and capital investment. ART utilization has been steadily rising, now with multiple available treatment platforms, but there is no generally accepted guideline for proper billing and reimbursement. No specific billing code for ART is currently available. Considering the heterogeneity of billing practices and limited published data on utilization trends, the American College of Radiation Oncology (ACRO) Government Relations and Economics Committee developed a survey to understand current clinical and billing practices while gauging the opinions of the community about appropriate reimbursement.

Methods: A two-part 21-item questionnaire was developed by ACRO and distributed amongst its members who are practicing attendings. The first section was intended for all attendings, including non-users of ART, and focused on opinions surrounding reimbursement, while the second section was meant only for ART-users, focusing on current billing practices and clinical utilization. Responses were collected between January 27, 2025 and April 1, 2025 and responses are reported with frequency counts and percentages.

Results: A total of 123 responses were collected, 51 were ART-users. A majority of respondents felt that a new billing code for ART should be developed (n=106; 87.8%) and a majority of respondents (n=101; 82.1%) either agreed or strongly agreed with the statement “Adaptive RT treatments should be reimbursed more than standard RT treatments.” Users are treating the full spectrum of disease with ART and using it for standard fractionation, hypofractionation and Stereotactic Body Radiation Therapy (SBRT). Attending physicians were almost universally involved in daily contouring for target volumes (n=46; 93.9%). Most users felt that their ART cases are generally more complex compared to non-ART cases (n=41; 83.7%) and proceeded with adaptation due to either changes in tumor volume (n=44; 89.8%) or changes in organs-at-risk (OARs) (n=40; 81.6%). Billing practices varied among ART-users and many respondents were unaware of which billing codes were being utilized (n=29; 59.2%).

Conclusion: This survey represents the first published material on the current clinical landscape and reimbursement practices of ART. ART is being used in many different ways and while current billing and clinical practices vary, the general opinion within the radiation oncology community is that ART should be reimbursed more than standard radiation treatments and we could benefit from the development of a unique billing code.

## Introduction

Delivery of radiation therapy (RT) has evolved significantly over the last half century, leading to more accurate treatment delivery, higher biological effective dose, and improved toxicity profiles. Technological advancements have improved our ability to immobilize patients and improve reproducibility, while upgrades in radiology and daily imaging have allowed us to better delineate the treatment target and surrounding organs-at-risk (OARs). However, modifying the planned treatment to conform to daily anatomic variation and tumor response during the course of treatment remains a challenge.

To address this challenge, adaptive radiation therapy (ART) was first proposed and adopted in 1997. ART introduced a process of adjusting the radiation plan based on changes observed in the tumor or nearby OARs throughout the course of RT [1]. Prior to significant technological innovation, “offline” ART was commonly performed by identifying systematic or progressive changes in the target and anatomy. In turn, a new plan is created, most often via the same clinical workflow as the initial treatment planning, with or without a new CT simulation scan depending on daily image quality, and the new plan is delivered for the remainder of the treatment course or until the next modification is required [2]. ART has been demonstrated to improve dosimetry across many disease sites, minimizing dose to OARs and improving coverage to targets, although data showing a clinical benefit is less abundant [3-6]. 

“Online” ART is the process of adjusting the treatment plan immediately prior to initiating treatment at the treatment console after evaluating the daily imaging, while the patient remains in the treatment position [7]. This form of ART has become increasingly popular and can require significant time from both the physician and the ancillary RT staff members. Online ART is commonly available in both CT-guided and MRI-guided platforms, requiring significant capital investment for acquisition, implementation and operation for this technology.

“Real-time” ART is a newer variation of ART, designed to manage intra-fraction changes and adjustment of the treatment plan during the actual delivery of RT. Some ART platforms, such as the ViewRay MRIdian™ system (Viewray Systems, Inc., USA) and Elekta Unity™ (Elekta AB, Sweden), incorporate both online ART and real-time ART.

While online and real-time ART have been available for over 10 years and utilization has been rising significantly, there is no current standard for ART reimbursement. The survey was developed to better understand the current clinical landscape as seen by ART users, with regard to practice patterns and reimbursement standards, while also gathering opinions from practicing radiation oncologists, including non-users of ART, to gauge the perceived value of and appropriate reimbursement for ART.

## Materials and methods

Survey construction

A 21-item anonymous questionnaire consisting of two sections was created based on discussion of American College of Radiation Oncology (ACRO) Government Relations and Economics Committee leadership and trainees. The first section, with seven items, was designed to gauge opinions of all respondents, including both users and non-users of ART. An eighth item asked respondents to select which ART machine they use in clinic, if no machine was selected, they were considered non-users of ART, and their survey was complete. Respondents who selected a listed machine, or “other,” indicated they were users of ART and thus continued on to the second section with an additional 13 items, which were designed to gather information on current clinical practices and reimbursement methods. The questionnaire was newly designed, with no prior testing or validation, and while the survey was edited thoroughly prior to distribution, no revisions took place after data collection began. The survey questions are available for review in the Appendix.

Section 1: Opinions

Respondents were asked their opinions regarding which ART modality provides the greatest, if any, benefit to the patient. Opinions on developing a new billing code for ART and whether ART treatments should be reimbursed more or less than standard RT treatments were also assessed. Appropriate relative reimbursement for ART for a single fraction compared to standard treatment planning codes were explored along with a full course of treatment with ART relative to standard reimbursement, with both queries split into professional and technical components.

Section 2: Clinical Utilization and Billing Practices

Users of ART were asked which ART system they use in clinic, the disease sites they treat with ART, the fractionation schemes used for ART (standard, hypofractionation, SBRT, etc.), number of patients beginning ART each week, and the percentage of physicians in their practice who utilize ART. Respondents were asked which team members were responsible for contouring OAR and target volumes. Participants were queried about their patient selection for ART, their use of prospective clinical trials for ART patients, the clinical indication for proceeding with daily adaptation, and whether the ART patients were generally of higher complexity. For billing, respondents were asked which billing codes they were currently using and whether they had restrictions on the maximum number of sessions billed.

Study population

Survey responses were collected between January 27, 2025 and April 1, 2025. The goal was to survey as many practicing radiation oncologists within the U.S. The survey could only be completed once by each respondent.

Using specialty specific data files from the American Medical Association (AMA) and ACRO, a list of 864 email address were identified and the survey was sent via email.

Email invitations to complete the questionnaire were distributed through the survey platform SurveyMonkey™ (Momentive, USA). Unique hyperlinks were sent to each email to prevent duplicative responses. Personally identifiable information was omitted from the data collection process to preserve respondent anonymity. An additional eight reminder emails were sent at varying intervals throughout the collection period to accounts that had not completed the survey.

A web link was also circulated via social media and distributed amongst colleagues to expand access, all responses from the web link were individually verified to ensure no respondent completed the survey more than once.

Data analysis

All responses were independently verified to ensure accurate reporting. All survey responses were summarized with frequency counts and percentages. No additional statistical analysis was required other than calculating the mean and median.

## Results

Of the 864 emails distributed, 111 (12.8%) were either returned with error codes or sent to accounts that opted out of receiving surveys. Of the remaining 753 emails, 109 responses (14.5%) were collected. An additional 14 responses were collected via the web link, therefore, there were 123 responses (16.3%) in total.

Of the 123 respondents, 72 (58.5%) were non-users of ART and only completed the first section, while 51 (41.5%) respondents were ART users. Two of the responses from ART users were considered incomplete as the respondent indicated they were an ART user but did not proceed to Section 2, therefore, for Section 1 there are 51 analyzable responses from ART users, but for Section 2, there are only 49 analyzable responses.

Opinions

Which Adaptive Modality Provides the Greatest Benefit to the Patient? (If >1 Modality Equivalent, Select All That Apply)

A majority of participants felt that CT-guided ART (n=70; 56.9%) was among the modalities that provided the greatest benefit to patients, while MRI-guided ART (n=60; 48.8%) and positron emission tomography (PET)-guided (n=17; 13.8%) less frequently selected. Of note, only three respondents (2.4%) believe there is no benefit to ART, while 27 participants (22.0%) felt they did not have enough experience with ART to select a modality.

Should a New Billing Code Be Created for Adaptive RT Treatments, Rather Than Utilizing Current IMRT Treatment Planning Codes (77301)?

Most respondents believe that a new billing code for ART should be created (n=106; 87.8%).

Adaptive RT Treatments Should Be Reimbursed More Than Standard RT Treatments

**Figure 1:**
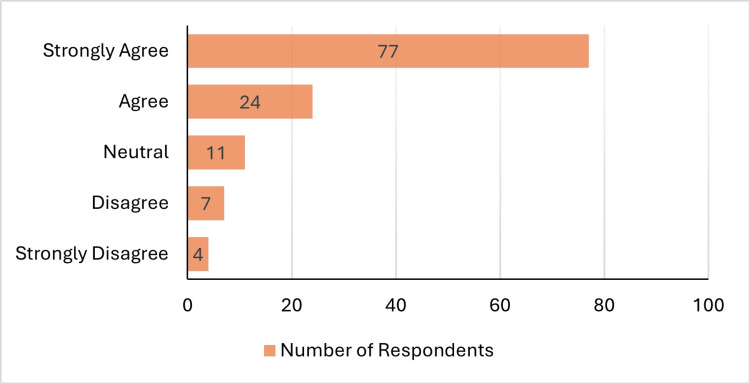
Opinions on higher reimbursement for ART ART: Adaptive radiation therapy

Most users either “Strongly Agreed” or “Agreed” about higher reimbursement (Figure 1). ART users were more likely to “Strongly Agree” (n=43; 84.3%) while non-users were not as convinced (n=34; 47.2%). All four respondents who strongly disagreed with the statement were non-users of ART.

Questions 4-7: Compared to Standard IMRT Treatment Planning Codes (77301), How Much Should Each Adaptive Treatment Planning Session Reimburse? (Q4-Professional Charges (PC), Q5-Technical Charges (TC)); The TOTAL Reimbursement for PROFESSIONAL(Q6) or TECHNICAL (Q7) Charges for a 5-Fraction Adaptive SBRT Treatment Should Be How Much More Than the Same Case Using Non-adaptive SBRT?

Table 1 presents the results.

**Table 1:** Results for Questions 4-7 ART: Adaptive radiation therapy; PC: Professional charges; TC: Technical charges

	All Respondents (n=123)	ART Non-Users (n=72)	ART Users (n=51)	ALL CT-Guided Users (n=35)	ALL MRI-Guided Users (n=16)
Q4 (PC) - mean (SD)	62.7% (34.1)	55% (33.8)	73.5% (31.8)	71.5% (32.5)	82.8% (23.4)
Q5 (TC) - mean (SD)	59.1% (35)	54% (33.5)	66.5% (36.2)	64.4% (36.5)	78.4% (29)
Q6 (PC) - mean (SD)	63.3% (34.1)	55.8% (32.9)	73.9% (33.2)	73.9% (31.6)	88.3% (17.7)
Q7 (TC) - mean (SD)	57.4 (34)	53% (30.9)	63.7% (37.3)	66.7% (34.8)	76.5% (31.4)

Which Adaptive RT Machine Do You Have in Clinic? (Select All That Apply)

Among ART users, the most used machine was the CT-based Varian Ethos™ (n=35; 68.6%), followed by ViewRay MRIdian™ (n=10; 19.6%) and Elekta Unity™ (n=6; 11.8%). Of note, eight respondents indicated they used more than one ART machine, and nine respondents indicated they used ART machines other than the three listed.

Clinical utilization and billing practices

Which Disease Sites Are You Treating With Adaptive Radiation? (Select All That Apply)

ART is being utilized for every definitive RT disease site along with oligometastatic indications, benign conditions and for palliation. The most commonly treated disease sites include lung (n=27; 55.1%), prostate (n=27; 55.1%), GYN (n=21; 42.9%), liver (n=21; 42.9%), and pancreas (n=21; 42.9%).

Which Types of Treatments Are You Adapting? (Select All That Apply)

Hypofractionation (n=37; 75.5%) and SBRT (n=35; 71.4%) are used more frequently than standard fractionation (n=29; 59.2%).

Approximately How Many New Patients Do You Plan to Start on Adaptive Radiation Each Week?

Among participants, the number of new patients starting each week ranged from 1-20, with a mean of 4.5 and median of 4.

Approximately What Percentage of Physicians in Your Practice Use Adaptive RT?

On average, the participants in our survey work in clinics where 52.8% of practicing physicians utilize ART.

Who Is Responsible for Daily Contouring of Organs-At-Risk? (Select All Involved)

There was significant heterogeneity in the responses to this question. Most frequently involved with OAR contouring were attending physicians (n=34; 69.4%) and auto-segmentation (n=33; 67.3%). Less involved were physics/dosimetry (n=11; 22.4%), resident physician (n=9; 18.4%) and therapists (n=2; 4.1%).

Who Is Responsible for Daily Contouring of Target Volumes? (Select All Involved)

There was less heterogeneity with contouring target volumes with most including an attending physician (n=46; 93.9%). Auto-segmentation (n=16; 32.7%), resident physician (n=7; 14.3%) and physics/dosimetry (n=2; 4.1%) were used less frequently for target volume contouring than for OAR contouring. Therapists were not used at all to contour target volumes.

How Do You Select Which Patients Are Treated on Adaptive Machine? (Select All That Apply)

A majority of respondents use “clinical evaluation based on anatomic features of case” (n=45; 91.8%) to determine which patients should receive ART. A handful of respondents treat “all patients of certain disease sites” (n=4; 8.2%) with ART without clinical evaluation of the case, while most others who selected this response also use clinical evaluation (n=12; 24.5%). Some respondents indicated they use predictive modeling as well (n=4; 8.2%).

What Is the Clinical Indication to Proceed With Daily Adaptation? (Select All That Apply)?

A majority of respondents used “tumor volume change” (n=44; 89.8%) and/or “OAR volume change" (n=40; 81.6%) to select when to proceed with daily adaptation. “Improvement in dosimetry” (n=26; 53.1%), “other anatomic changes or changes in setup” (n=24; 49%) and “set schedule to adapt after a certain number of fractions” (n=5; 10.2%) were used less frequently to influence the decision to proceed with daily adaptation.

Which Billing Codes Are You Submitting for an Adaptive Treatment?

A majority of respondents are unsure (n=29; 59.2%) which billing codes are being submitted for an adaptive treatment while others are using 77301 (n=19; 38.8%). One respondent indicated they do not bill for ART.

For SBRT Is There a Maximum Number of Adaptive Sessions Billed Per Treatment Course?

Some respondents indicated there is no limit and can bill up to 5 times (n=19; 38.8%) while fewer respondents said they were restricted in billing either by “time interval (ex: once a week)” (n=5; 10.2%) or restricted by “number of sessions (ex: maximum of 3 times)” (n=4; 8.2%). Many respondents are not aware of their clinic’s billing practices (n=21; 42.9%).

For Non-SBRT (>5 Fractions) Is There a Maximum Number of Adaptive Sessions Billed Per Treatment Course?

Again, 21 respondents are not aware of their clinic’s billing practices (42.9%), however, fewer selected “they can bill each fraction without limitation” (n=7; 14.3%). More frequently respondents indicated they are restricted with how frequently they bill based on a total number of sessions (n=13; 26.5%) or by time interval (n=8; 16.3%).

Do You Have Open Prospective Clinical Trials for Your Adaptive Patients?

A majority of respondents indicated they do not have open prospective clinical trials (n=29; 59.2%). Of the respondents who do have open prospective clinical trials (n=20; 40.8%), disease sites for the open trials include Anus, Breast, CNS, Head and Neck, Cervix, Prostate, Pancreas, abdominal metastases and oligometastases. 

In Your Experience, Are Your Adaptive Patients Generally Higher Complexity Cases (High Dose Per Fraction, Re-RT etc.) Compared to Non-Adaptive Patients?

A majority of respondents believe that their ART patients are generally more complex (n=41; 83.7%).

## Discussion

This survey represents the first published report to characterize the current clinical landscape and reimbursement practices of ART while also capturing the prevailing attitudes of radiation oncologists towards its use. Respondents demonstrated substantial variability in clinical exposure to ART; over half reported not actively utilizing ART in practice, whereas those who do employ ART indicated its application across a broad range of disease sites.

Respondents had a generally positive view of ART, with only 2.4% indicating there is no benefit of ART. Furthermore, over 80% of respondents either agreed or strongly agreed that ART treatments should be reimbursed more than standard RT, while only 9% disagreed or strongly disagreed. There was consensus that a new billing code should be created for ART, with 88% of respondents in agreement.

From a reimbursement standpoint, respondents believed a single ART planning session should reimburse at least 50% of the current intensity-modulated radiation therapy (IMRT) treatment planning codes, and for a 5-fraction SBRT with ART, total reimbursement should be at least 50% more than the same plan without ART, both for professional and technical components. Unsurprisingly, ART users felt ART reimbursement should be higher than non-users. Furthermore, users of MRI-guided ART felt more strongly about higher reimbursements than CT-guided ART users. This is not unexpected considering the greater time required for MRI-guided ART and the increased capital investment [4].

There was no consensus regarding which ART modality provides the greatest benefit, with CT-guided and MRI-guided ART being the most popular responses. The differences in opinion between users of CT-guided versus MRI-guided ART could spark debate about whether a new billing code for ART should value CT-guided and MRI-guided ART differently. Disease-specific evidence for both modalities exist on their own, but there are no published direct comparisons between the two modalities [4,8].

A key finding of this survey is the broad range of disease sites and clinical indications for which ART is currently being used. At least five respondents indicated they are using ART for each disease site listed in our survey. There was notable heterogeneity in fractionation strategies as more than 50% of respondents reported using ART with SBRT, hypofractionation and standard fractionation schedules. Even though current evidence for most disease sites is limited to a dosimetric benefit, these survey results suggest widespread belief in the potential benefits across all disease sites [3-8].

The survey attempted to elicit whether auto-segmentation, resident physicians, attending physicians or others are involved in contouring OARs and target volumes. Of note, less than 5% respondents indicated that therapists were involved with contouring OARs and no respondents involved therapists for target delineation. Attending physicians were much more likely to be involved with contouring target volumes than OARs (94% vs 69%). These observations are consistent with the recently published American Society for Radiation Oncology (ASTRO) White Paper that states that at a minimum, there should be physician oversight with contouring, especially for target volumes [9]. These results are concordant with the majority belief that ART should be reimbursed more than standard RT, considering the increased physician efforts. Defining appropriate workflow and roles of team members will be an important issue to standardize if an ART billing code were to be pursued. 

ART patient volume was modest with an average of 4.5 new patients per week, with only five respondents indicating an average of 10 or more new starts per week. Most ART users are using “clinical evaluation based on anatomic features of a case” to determine which patients should be treated with ART and are using either “tumor volume or OAR volume change” to select when to proceed with daily adaptation. Again, this is consistent with the recent ASTRO white paper in terms of the most appropriate way to triage which patients would benefit from ART [9]. When considering the development of a billing code, there will likely need to be guidelines defining eligible patients and other indications for ART. Regardless of whether it will be for certain disease sites, specific fractionation schemes or something else, it will be important to appropriately define the indications should a billing code be developed.

Another notable and potentially concerning finding from our survey is the widespread lack of awareness amongst respondents regarding their current billing practices, with 59% of respondents indicating they were unsure of how ART was billed at their institutions. Amongst those respondents who were familiar with their billing protocols, there was considerable variability in practice with some reporting no limitations in the number of ART sessions billed, while others noted restrictions based on time or number of fractions. The implementation of a dedicated ART code could help standardize reimbursement practices by either enabling billing across all ART sessions or alternatively, establishing clear uniform guidelines outlining allowable use. Another concern raised by the survey results is the lack of open clinical trials among ART users, with only 41% of users participating in prospective clinical trials and many treated disease sites without open prospective clinical trials. To our knowledge, there is no published phase III data or open phase III trials specifically evaluating online or real-time ART and it is critical that ART users consider opening trials to help build a foundation of data which can ultimately be used as evidence to define and refine indications for ART.

This survey has limitations that should not be ignored. All respondents received the survey either directly by email or heard about it from ACRO’s social media efforts, or by word of mouth, likely via an ACRO member. Participants of this survey were all members of a single organization, which could lead to questions of the external validity of the survey. Therefore, it is our hope that this survey is viewed simply as a first attempt to understand the landscape of ART. Any attempts at policy changes should be guided by more comprehensive surveys and inclusion of broader representation of the specialty. 

Another potential limitation of the survey is that a few “users” who selected “other” as their ART system described using equipment or processes most consistent with offline ART. While the opinions of offline ART users are still valuable, the survey was more intended to survey those using online ART and real-time ART considering the additional technological and financial investment required to provide those services. Offline ART can be done on most modern RT machines and therefore may not require changes to current reimbursement practices. Should a new billing code for ART be developed, the distinction of which forms of ART are inclusive will be critically important. 

## Conclusions

This survey offers important preliminary insights into the rapidly evolving clinical landscape of ART and its current clinical landscape. The current utilization patterns demonstrate that ART is being used across the wide range of disease sites. The absence of robust clinical trials data and inconsistencies of billing practices ART highlight possible gaps in standardization with potential impact on quality of care. If an ART-specific billing code is to be implemented as favored by the majority of our survey respondents, it must be accompanied by a coordinated effort to generate high-quality evidence, delineate appropriate clinical indications, define staffing roles and conduct a broader workforce assessment to guide policy and practice. Implementation of a billing code along with a coordinated national effort could help increase availability of ART nationwide. Overall, ART is generally perceived as clinically advantageous with strong support for incremental reimbursement for professional and technical services relative to conventional, non-adaptive planning.

## Appendices

Opinions

1. Which adaptive modality provides the greatest benefit to the patient? (if >1 modality equivalent, select all that apply)

·       CT-based ·       MRI-based ·       PET-guided ·       No benefit with ART ·       Not enough experience with ART to say ·       Other (please specify)

2. Should a new billing code be created for adaptive RT treatments, rather than utilizing current IMRT treatment planning codes (77301)?

·       Yes ·       No

3. Adaptive RT treatments should be reimbursed more than standard RT treatments

·       Strongly agree ·       Agree ·       Neutral ·       Disagree ·       Strongly disagree

4. Compared to standard IMRT treatment planning codes (77301), how much should each adaptive treatment planning session reimburse? (Professional charges)

·       0% (no additional reimbursement for adaptive treatment) - 100% (same as 77301)

5. Compared to standard IMRT treatment planning codes (77301), how much should each adaptive treatment planning session reimburse? (Technical charges)

·       0% (no additional reimbursement for adaptive treatment) - 100% (same as 77301)

6. The TOTAL reimbursement for PROFESSIONAL charges for a 5-fraction adaptive SBRT  treatment should be how much more than the same case using non-adaptive SBRT?

·       0% (same) - 100% (twice as much)

7. The TOTAL reimbursement for TECHNICAL charges for a 5-fraction adaptive SBRT treatment should be how much more than the same case using non-adaptive SBRT?

·       0% (same) - 100% (twice as much)

8. Which adaptive RT machine do you have in clinic? (select all that apply)

·       Varian Ethos ·       ViewRay MRIdian ·       Elekta Unity ·       Elekta Evo ·       Reflexion ·       Other (please specify) ·       None of the above

Clinical utilization and billing practices

9. Which disease sites are you treating with adaptive radiation? (select all that apply)

·       Anorectal ·       Bladder ·       CNS ·       GYN ·       Head & neck ·       Kidney ·       Liver ·       Lung ·       Pancreas ·       Prostate ·       Palliative ·       Other (please specify)

10. Which types of treatments are you adapting? (select all that apply)

·       Standard fractionation ·       Hypofractionation ·       SBRT

11. Approximately how many new patients do you plan to start on adaptive radiation each week?

·       0 - 20

12. Approximately what percentage of physicians in your practice use adaptive RT?

·       0 - 100

13. Who is responsible for daily contouring of Organs-At-Risk? (select all involved)

·       Auto-segmentation ·       Resident physician ·       Attending physician ·       Other (please specify)

14. Who is responsible for daily contouring of target volumes? (select all involved)

·       Auto-segmentation ·       Resident physician ·       Attending physician ·       Other (please specify)

15. How do you select which patients are treated on adaptive machine? (select all that apply)

·       Clinical trial patients ·       Clinical registry ·       All patients of certain disease sites ·       Clinical evaluation based on anatomic features of case ·       Predictive Modeling of patients to select most likely to benefit from adaptation ·       Other (please specify)

16. What is the clinical indication to proceed with daily adaptation? (select all that apply)?

·       Tumor volume change ·       OAR location change ·       Improvement in dosimetry ·       Other anatomic changes or changes in setup ·       Set schedule to adapt after a certain number of fractions ·       Other (please specify)

17. Which billing codes are you submitting for an adaptive treatment?

·       77301 ·       Unsure ·       Other (please specify)

18. For SBRT is there a maximum number of adaptive sessions billed per treatment course?

·       No, can bill up to 5 times ·       Yes, restricted by time interval (ex: once a week) ·       Yes, up to a certain number of total sessions (ex: maximum of 3 times) ·       Other (please specify)

19. For non-SBRT (>5 fractions) is there a maximum number of adaptive sessions billed per treatment course?

·       No, can bill each fraction without limitation ·       Yes, restricted by time interval (ex: once a week) ·       Yes, up to a certain number of total sessions (ex: maximum of 5 times) ·       Other (please specify)

20. Do you have open prospective clinical trials for your adaptive patients?

·       No ·       Yes (If yes, please specify which disease sites)

21. In your experience, are your adaptive patients generally higher complexity cases (high dose per fraction, re-RT etc) compared to non-adaptive patients?

·       Yes ·       No
